# National Pharmacovigilance Assessment of Oral Adverse Events Following COVID-19 Vaccination in Germany (2020-2023)

**DOI:** 10.1016/j.identj.2025.100906

**Published:** 2025-07-19

**Authors:** Abanoub Riad

**Affiliations:** Masaryk Centre for Global Health (MCGH), Department of Public Health, Faculty of Medicine, Masaryk University, Brno, Czech Republic; Department of Public Health, Faculty of Medicine, Masaryk University, Brno, Czech Republic

**Keywords:** Adverse Drug Reaction Reporting Systems, COVID-19 vaccines, Germany, Oral manifestations, Pharmacovigilance

## Abstract

**Objectives:**

Pharmacovigilance efforts for COVID-19 vaccines have largely focused on severe adverse events (AEs), while nonserious, yet distressing, AEs, such as oral AEs, remain underexamined. This study aimed to analyse oral AE reporting patterns in the German national pharmacovigilance database.

**Methods:**

A retrospective analysis of individual case safety reports (ICSRs) from the Paul-Ehrlich-Institut (PEI) database was conducted for December 2020 to December 2023. The absolute reporting ratio was calculated as cases per 1000 ICSRs for each oral AE. Secondary analyses included: (1) cross-database comparisons with the U.S. Vaccine Adverse Event Reporting System (VAERS); (2) disproportionality analysis using a hybrid approach combining frequentist and Bayesian conditions to establish signals of disproportionate reporting (SDRs); (3) subgroup analyses based on demographic and vaccine-related factors; and (4) multivariable regression to adjust for potential confounders.

**Results:**

Gustatory AEs, such as ageusia and dysgeusia; other oral sensory AEs, including oral paraesthesia and oral hypoaesthesia; and specific mucosal AEs, such as oral herpes and aphthous stomatitis, were the most frequently reported oral AEs in the PEI dataset. Cross-database analysis not only confirmed the prominence of gustatory and other sensory AEs but also highlighted differences, with VAERS reporting higher rates of swollen tongue and lip swelling. Disproportionality analysis identified 21 oral AEs as true SDRs. Female susceptibility was evident in several oral AEs, and age-stratified analysis revealed higher reporting among minors and seniors compared to middle-aged adults. Differences in oral AE reporting between mRNA and viral vector vaccines lacked a consistent pattern, and booster doses were associated with increased reporting of select oral AEs.

**Conclusions:**

Within the limitations of passive surveillance data, this study highlights the need for further research on oral AEs using self-controlled case-series designs for clinically significant events. Integrating oral AEs into vaccine safety monitoring could improve postmarketing surveillance, while validated AEs may warrant inclusion in product information for transparency.

## Introduction

The safety of COVID-19 vaccines has been extensively investigated through global pharmacovigilance systems, primarily focusing on severe adverse events (AEs) such as myocarditis, thromboembolic events, and anaphylaxis.^1^, ^2^, ^3^ However, nonsevere, yet potentially distressing, AEs, including those affecting the oral cavity, remain underexplored. Oral AEs, such as taste disturbances, mucosal lesions, and swelling of the lips or tongue, have been sporadically reported in postmarketing surveillance, yet their epidemiological patterns and clinical relevance are not well established.^4^^,^^5^ Given the widespread administration of COVID-19 vaccines and the essential role of oral health in overall well-being, systematic investigations into oral AEs are necessary to better characterise their frequency, nature, and potential implications.^6^, ^7^, ^8^

Pharmacovigilance databases provide valuable insights into vaccine safety by capturing real-world AE reports from healthcare professionals and vaccine recipients.^9^ Among these, the German Paul-Ehrlich-Institut (PEI) pharmacovigilance system offers a unique opportunity to examine oral AEs following COVID-19 vaccination in a European context.^10^ With over 190 million COVID-19 vaccine doses administered in Germany between December 2020 and December 2023, the PEI database is distinct in that each individual case safety report (ICSR) is assigned a single ‘chief complaint’, which may influence AE reporting patterns compared to other national pharmacovigilance systems.^11^ Understanding these structural differences is essential for contextualising findings within global vaccine safety assessments.

Signal detection in pharmacovigilance relies on disproportionality analysis to identify AEs reported at unexpectedly high frequencies relative to the overall dataset.^12^, ^13^, ^14^ Traditional methods, such as the proportional reporting ratio (PRR) and reporting odds ratio (ROR), are widely used but may generate spurious associations due to statistical artefacts or reporting biases.^14^ To enhance specificity and minimise false-positive signals, a hybrid approach incorporating Bayesian methods, such as the information component (IC), has gained recognition in postmarketing safety surveillance.^13^ Employing a hybrid disproportionality analysis allows for the identification of true signals of disproportionate reporting (SDRs) while mitigating the risk of false alarms.

The primary objective of this study is to characterise the spectrum and reporting patterns of oral AEs following COVID-19 vaccination in the German PEI dataset. Secondary objectives include^1^: validating findings through cross-database comparison with the U.S. Vaccine Adverse Event Reporting System (VAERS)^2^; analysing demographic and vaccine-specific reporting trends through stratification by sex, age group, vaccine type, and vaccination schedule; and^3^ employing multivariable regression to control for potential confounding variables.

## Materials and methods

### Study design

A retrospective analysis was performed using ICSRs from the PEI pharmacovigilance database covering the period from December 2020 to December 2023.^11^ The present study analysed oral AEs following COVID-19 vaccination and complied with the REporting of A Disproportionality Analysis for DrUg Safety Signal Detection Using Individual Case Safety Reports in PharmacoVigilance guidelines to ensure methodological rigour and transparency.^9^

### Data source

The PEI dataset of suspected side effects following COVID-19 vaccination encompasses ICSRs from healthcare professionals, vaccinated individuals, pharmaceutical companies, and regulatory bodies, submitted via mail, email, phone, the PEI online portal, and the European Medicines Agency EudraVigilance database.^11^ Reporting obligations for medical professionals are regulated by national laws, with cases first reported to professional drug commissions or public health departments before being forwarded to PEI.^15^ Each ICSR records only a single clinical symptom ‘chief complaint’, and duplicate ICSRs from multiple sources were merged to ensure accurate safety signal detection at the federal level.^11^

### Variables

A previously described anatomo-physiological framework of the oral cavity was utilised, classifying AEs according to anatomical regions (lips, palate, tongue, teeth, salivary glands, oral mucosa) and functions (taste and other sensations).^6^ Relevant keywords associated with this framework were searched in the PEI dataset, while terms unrelated to the oral cavity – such as oral contraception, congenital conditions (eg, cleft lip and palate), and oncologic disorders (eg, stage II tongue cancer) – were excluded due to biological implausibility and their extremely low frequency (*n* < 3). A final list of 80 oral AEs was identified in the PEI dataset and subsequently analysed in this study.

### Statistical analyses

Statistical analyses were conducted in two stages. Primary analysis assessed the absolute reporting ratios (ARRs) for each oral AE in the PEI dataset. Secondary analyses aimed to control for potential reporting biases and disproportionality, despite the absence of comparator data (other vaccines/drugs) in the PEI dataset. This was achieved through four steps:

1. *Cross-database Analysis*: ARRs of oral AEs in the PEI dataset were compared with corresponding values from the VAERS (data extracted in October 2024).^16^ The odds ratio (OR) of PEI to VAERS, along with 95% CI, was calculated to highlight disparities in reporting trends.
2. *Disproportionality Analysis*: SDRs were identified using VAERS data. Oral AEs were assessed based on frequentist disproportionality metrics – the PRR and ROR – as well as a Bayesian method using the IC. AEs meeting all three criteria were flagged as SDRs.^13^
3. *Subgroup Analysis*: ARRs for the 20 most commonly reported oral AEs in the PEI dataset were stratified by sex (female vs male), age group (minors <18, adults 18-59, and seniors >59), year of submission (2020, 2021, 2022, 2023), vaccine type (mRNA, viral vector, and protein subunit), and vaccine schedule (primary vs booster series).
4. *Regression Analysis*: Multivariable logistic regression was performed for each of the top 20 AEs, with 4 predictors: sex, age group, vaccine type, and vaccine schedule.

All analyses were conducted using RStudio (Version 2024.12.1+563; Posit PBC) and IBM SPSS Statistics (Version 29; IBM Corp.) with a significance level of *P* < .05.

## Results

The PEI dataset comprised 974,931 ICSRs, with reports most frequently submitted in 2021 (61.71%), followed by 2022 (32.56%), 2023 (5.72%), and 2020 (0.01%). Compared to the VAERS dataset (1,016,024 ICSRs), the PEI dataset had a higher proportion of female reports (71.98% vs 66.50%; *P* < .001) and more adults aged 18-59 years (82.59% vs 56.65%; *P* < .001). It also included fewer reports from mRNA vaccine recipients (76.88% vs 92.73%) but a higher proportion from viral vector vaccine recipients (22.74% vs 7.22%; *P* < .001), as well as more reports associated with primary series doses (99.35% vs 95.99%; *P* < .001) (Table 1).

**Table 1:** Characteristics of individuals reporting adverse events following COVID-19 vaccination in the Paul-Ehrlich-Institute (PEI) dataset (Germany, December 2020-December 2023) and VAERS dataset (United States, December 2020-October 2024).

Variable	Outcome	PEI, Germany (ICSR, *N* = 974,931)	VAERS, USA (ICSR, *N* = 1,016,024)	*P*
Sex	Female	694,572 (71.98%)	647,009 (66.50%)	**<.001**
	Male	270,362 (28.02%)	325,929 (33.50%)	
	*Missed*	9997 (1.03%)	43,086 (4.24%)	
Age group	<2 y	66 (0.01%)	2807 (0.31%)	**<.001**
	2-6 y	801 (0.09%)	8917 (0.98%)	
	7-17 y	16,129 (1.72%)	51,139 (5.61%)	
	18-59 y	773,889 (82.59%)	516,801 (56.65%)	
	>59 y	146,154 (15.6%)	332,564 (36.46%)	
	*Missed*	37,892 (3.89%)	103,796 (10.22%)	
Vaccine type	mRNA	745,174 (76.88%)	939,716 (92.73%)	**<.001**
	Viral vector	220,371 (22.74%)	73,201 (7.22%)	
	Protein subunit	3658 (0.38%)	511 (0.05%)	
	Inactivated virus	18 (<0.01%)	*NA*	
	*Missed*	5710 (0.59%)	2596 (0.26%)	
Vaccine schedule	Primer series	962,881 (99.35%)	972,752 (95.99%)	**<.001**
	Booster series	6340 (0.65%)	40,676 (4.01%)	
	*Missed*	5710 (0.59%)	2596 (0.26%)	
Year	2020	113 (0.01%)	10,380 (1.02%)	**<.001**
	2021	601,610 (61.71%)	710,616 (69.94%)	
	2022	317,453 (32.56%)	210,534 (20.72%)	
	2023	55,755 (5.72%)	71,074 (7.00%)	
	2024	*NA*	13,420 (1.32%)	

ICSR, individual case safety report; VAERS, U.S. Vaccine Adverse Event Reporting System.

Chi-squared (χ^2^) test was used with a significance level of *P* < 0.05.

### Primary analysis

Taste disorder had the highest ARR (0.493 cases per 1000 ICSRs), followed by ageusia ‘complete loss of taste’ (0.449), oral herpes (0.411), dysgeusia ‘altered taste’ (0.361), oral paraesthesia ‘tingling or numbness’ (0.325), oral hypoaesthesia ‘reduced sensitivity of mouth’ (0.319), swollen tongue (0.297), dry mouth (0.277), lip swelling (0.258), and aphthous ulcer (0.129) according to the PEI dataset (Table 2).

**Table 2:** Oral adverse events following COVID-19 vaccination in the Paul-Ehrlich-Institute (PEI) dataset (Germany; December 2020-December 2023) and the VAERS dataset (United States; December 2020-October 2024).

Preferred term	Group	PEI, Germany *N* (ARR) ▼	VAERS, USA *N* (ARR)	Disproportionality analysis of VAERS data				OR (95% CI) PEI vs VAERS
				PRR (95% CI)	ROR (95% CI)	IC (IC25)	SDR	
Taste disorder	Taste	481 (0.493)	296 (0.291)	17.57 (10.28-30.03)	17.57 (10.28-30.04)	4.14 (3.60)	Yes	1.69 (1.47-1.96)
Ageusia	Taste	438 (0.449)	3491 (3.436)	15.51 (13.39-17.97)	15.56 (13.43-18.03)	3.96 (3.81)	Yes	0.13 (0.12-0.14)
Oral herpes	Oral mucosa	401 (0.411)	433 (0.426)	1.36 (1.17-1.59)	1.36 (1.17-1.59)	0.45 (0.29)	No	0.97 (0.84-1.11)
Dysgeusia	Taste	352 (0.361)	2232 (2.197)	3.31 (3.02-3.63)	3.32 (3.02-3.64)	1.73 (1.64)	Yes	0.16 (0.15-0.18)
Paraesthesia oral	Sensation	317 (0.325)	1183 (1.164)	4.47 (3.87-5.16)	4.47 (3.87-5.16)	2.16 (2.02)	Yes	0.28 (0.25-0.32)
Hypoaesthesia oral	Sensation	311 (0.319)	1132 (1.114)	4.26 (3.69-4.92)	4.26 (3.69-4.92)	2.09 (1.95)	Yes	0.29 (0.25-0.32)
Swollen tongue	Tongue	290 (0.297)	584 (0.575)	2.70 (2.28-3.19)	2.70 (2.28-3.19)	1.43 (1.26)	Yes	0.52 (0.45-0.60)
Dry mouth	Salivary G.	270 (0.277)	626 (0.616)	2.59 (2.21-3.03)	2.59 (2.21-3.04)	1.37 (1.21)	Yes	0.45 (0.39-0.52)
Lip swelling	Lip	252 (0.258)	1721 (1.694)	1.54 (1.42-1.67)	1.54 (1.42-1.67)	0.62 (0.54)	No	0.15 (0.13-0.17)
Aphthous ulcer	Oral mucosa	126 (0.129)	275 (0.271)	4.48 (3.32-6.04)	4.48 (3.32-6.04)	2.16 (1.87)	Yes	0.48 (0.39-0.59)
Oral disorder	Oral mucosa	119 (0.122)	44 (0.043)	1.74 (1.04-2.93)	1.74 (1.04-2.93)	0.80 (0.28)	No	2.82 (1.99-3.98)
Tongue disorder	Tongue	111 (0.114)	72 (0.071)	1.62 (1.09-2.40)	1.62 (1.09-2.40)	0.69 (0.30)	No	1.61 (1.19-2.16)
Stomatitis	Oral mucosa	88 (0.09)	120 (0.118)	0.66 (0.52-0.83)	0.66 (0.52-0.83)	-0.61 (-0.85)	No	0.76 (0.58-1.01)
Hypogeusia	Taste	82 (0.084)	30 (0.03)	3.56 (1.56-8.11)	3.56 (1.56-8.11)	1.83 (1.01)	Yes	2.85 (1.88-4.33)
Mouth swelling	Oral mucosa	52 (0.053)	133 (0.131)	2.35 (1.69-3.28)	2.35 (1.69-3.28)	1.23 (0.90)	Yes	0.41 (0.30-0.56)
Hyper salivation	Salivary G.	35 (0.036)	0 (0)	*NA*	*NA*	*NA*	NA	∞
Glossodynia	Tongue	31 (0.032)	177 (0.174)	2.49 (1.86-3.35)	2.49 (1.86-3.35)	1.32 (1.02)	Yes	0.18 (0.12-0.27)
Oral mucosal eryth.	Oral mucosa	25 (0.026)	8 (0.008)	1.33 (0.44-4.06)	1.33 (0.44-4.06)	0.41 (-0.71)	No	3.26 (1.47-7.22)
Sialadenitis	Salivary G.	22 (0.023)	0 (0)	*NA*	*NA*	*NA*	NA	∞
Tongue coated	Tongue	20 (0.021)	2 (0.002)	*NA*	*NA*	*NA*	NA	10.42 (2.44-44.59)
Lip oedema	Lip	19 (0.019)	22 (0.022)	1.52 (0.75-3.08)	1.52 (0.75-3.08)	0.61 (-0.10)	No	0.90 (0.49-1.66)
Tongue paralysis	Tongue	15 (0.015)	2 (0.002)	0.33 (0.06-1.71)	0.33 (0.06-1.71)	-1.59 (-3.23)	No	7.82 (1.79-34.18)
Anaesthesia oral	Sensation	15 (0.015)	10 (0.01)	8.31 (1.06-64.91)	8.31 (1.06-64.91)	3.06 (1.00)	Yes	1.56 (0.70-3.48)
Oral mucosal blister	Oral mucosa	15 (0.015)	63 (0.062)	1.03 (0.71-1.49)	1.03 (0.71-1.49)	0.04 (-0.33)	No	0.25 (0.14-0.44)
Dental discomfort	Dentition	15 (0.015)	32 (0.031)	*NA*	*NA*	*NA*	NA	0.49 (0.26-0.90)
Oral dysaesthesia	Sensation	14 (0.014)	1 (0.001)	*NA*	*NA*	*NA*	NA	14.59 (1.92-110.96)
Cheilitis	Lip	14 (0.014)	201 (0.198)	1.51 (1.19-1.90)	1.51 (1.19-1.90)	0.59 (0.36)	No	0.07 (0.04-0.12)
Noninfective sialo.	Salivary G.	13 (0.013)	5 (0.005)	*NA*	*NA*	*NA*	NA	2.71 (0.97-7.60)
Tongue movement ds.	Tongue	13 (0.013)	6 (0.006)	4.99 (0.60-41.41)	4.99 (0.60-41.41)	2.32 (0.20)	No	2.26 (0.86-5.94)
Oral candidiasis	Oral mucosa	13 (0.013)	33 (0.032)	1.83 (0.99-3.37)	1.83 (0.99-3.37)	0.87 (0.26)	No	0.41 (0.22-0.78)
Lip dry	Lip	13 (0.013)	33 (0.032)	2.29 (1.18-4.42)	2.29 (1.18-4.42)	1.19 (0.53)	Yes	0.41 (0.22-0.78)
Glossitis	Tongue	12 (0.012)	49 (0.048)	1.40 (0.89-2.22)	1.40 (0.89-2.22)	0.49 (0.03)	No	0.26 (0.14-0.48)
Trichoglossia	Tongue	10 (0.01)	1 (0.001)	*NA*	*NA*	*NA*	NA	10.42 (1.33-81.41)
Palatal swelling	Palate	10 (0.01)	13 (0.013)	*NA*	*NA*	*NA*	NA	0.80 (0.35-1.83)
Aptyalism	Salivary G.	9 (0.009)	15 (0.015)	6.23 (1.43-27.25)	6.23 (1.43-27.25)	2.64 (1.16)	Yes	0.63 (0.27-1.43)
Tongue blistering	Tongue	9 (0.009)	19 (0.019)	3.16 (1.18-8.46)	3.16 (1.18-8.46)	1.66 (0.67)	Yes	0.49 (0.22-1.09)
Tongue eruption	Tongue	9 (0.009)	6 (0.006)	2.49 (0.50-12.35)	2.49 (0.50-12.35)	1.32 (-0.28)	No	1.56 (0.56-4.39)
Tongue oedema	Tongue	9 (0.009)	3 (0.003)	0.05 (0.02-0.17)	0.05 (0.02-0.17)	-4.24 (-5.40)	No	3.13 (0.85-11.55)
Oral mucosa erosion	Oral mucosa	9 (0.009)	1 (0.001)	*NA*	*NA*	*NA*	NA	9.38 (1.19-74.04)
Oral pruritus	Oral mucosa	9 (0.009)	112 (0.11)	2.39 (1.66-3.44)	2.39 (1.66-3.44)	1.26 (0.89)	Yes	0.08 (0.04-0.17)
Perioral dermatitis	Oral mucosa	9 (0.009)	1 (0.001)	0.83 (0.05-13.29)	0.83 (0.05-13.29)	-0.27 (-3.04)	No	9.38 (1.19-74.04)
Hyperaesthesia teeth	Dentition	9 (0.009)	44 (0.043)	9.14 (3.28-25.44)	9.14 (3.28-25.44)	3.19 (2.17)	Yes	0.21 (0.10-0.44)
Tongue discolouration	Tongue	8 (0.008)	27 (0.027)	1.40 (0.76-2.60)	1.40 (0.76-2.60)	0.49 (-0.13)	No	0.31 (0.14-0.68)
Oral lichen planus	Oral mucosa	8 (0.008)	8 (0.008)	3.32 (0.71-15.65)	3.32 (0.71-15.65)	1.73 (0.18)	No	1.04 (0.39-2.78)
Oral fungal infection	Oral mucosa	7 (0.007)	4 (0.004)	3.32 (0.37-29.74)	3.32 (0.37-29.74)	1.73 (-0.46)	No	1.82 (0.53-6.23)
Oral mucosal eruption	Oral mucosa	7 (0.007)	52 (0.051)	1.49 (0.95-2.35)	1.49 (0.95-2.35)	0.58 (0.12)	No	0.14 (0.06-0.31)
Salivary gland enlarg.	Salivary G.	6 (0.006)	11 (0.011)	1.31 (0.51-3.37)	1.31 (0.51-3.37)	0.39 (-0.56)	No	0.57 (0.21-1.54)
Saliva changes	Salivary G.	6 (0.006)	0 (0)	*NA*	*NA*	*NA*	NA	∞
Angular cheilitis	Lip	6 (0.006)	11 (0.011)	2.29 (0.73-7.18)	2.29 (0.73-7.18)	1.19 (0.05)	No	0.57 (0.21-1.54)
Lip blister	Lip	6 (0.006)	74 (0.073)	1.28 (0.89-1.84)	1.28 (0.89-1.84)	0.36 (-0.01)	No	0.08 (0.04-0.19)
Circumoral swelling	Oral mucosa	5 (0.005)	31 (0.031)	3.68 (1.62-8.36)	3.68 (1.62-8.36)	1.88 (1.06)	Yes	0.17 (0.07-0.43)
Tongue dry	Tongue	4 (0.004)	11 (0.011)	*NA*	*NA*	*NA*	NA	0.38 (0.12-1.19)
Tongue erythema	Tongue	4 (0.004)	13 (0.013)	3.60 (1.03-12.64)	3.60 (1.03-12.64)	1.85 (0.59)	Yes	0.32 (0.10-0.98)
Tongue ulceration	Palate	4 (0.004)	18 (0.018)	1.25 (0.60-2.59)	1.25 (0.60-2.59)	0.32 (-0.41)	No	0.23 (0.08-0.68)
Palatal disorder	Palate	4 (0.004)	3 (0.003)	2.49 (0.26-23.97)	2.49 (0.26-23.97)	1.32 (-0.95)	No	1.39 (0.31-6.21)
Lip disorder	Lip	4 (0.004)	33 (0.032)	3.92 (1.73-8.86)	3.92 (1.73-8.86)	1.97 (1.15)	Yes	0.13 (0.04-0.36)
Lip erythema	Lip	4 (0.004)	28 (0.028)	2.59 (1.22-5.48)	2.59 (1.22-5.48)	1.37 (0.62)	Yes	0.15 (0.05-0.42)
Lip pain	Lip	4 (0.004)	59 (0.058)	1.96 (1.23-3.13)	1.96 (1.23-3.13)	0.97 (0.50)	No	0.07 (0.03-0.19)
Chapped lips	Lip	3 (0.003)	128 (0.126)	2.53 (1.79-3.59)	2.53 (1.79-3.59)	1.34 (0.99)	Yes	0.02 (0.01-0.08)
Dental paraesthesia	Dentition	3 (0.003)	12 (0.012)	9.97 (1.30-76.69)	9.97 (1.30-76.69)	3.32 (1.28)	Yes	0.26 (0.07-0.92)

VAERS, U.S. Vaccine Adverse Event Reporting System.

The absolute reporting rate (ARR) represents the number of reported cases per 1000 individual case safety reports.

The conditions of establishing a signal of disproportionate reporting (SDR) are (i) proportional reporting ratio (PRR) ≥ 2 and its 95% CI lower bound ≥ 1, (ii) reporting odds ratio (ROR) ≥ 2 and its 95% CI lower bound ≥ 1, and iii) information component (IC) method point estimate and its IC25 > 0.

The following preferred terms were trimmed because they had fewer than three reports in the PEI dataset: salivary gland pain, stiff tongue, tongue exfoliation, tongue pruritus, tongue spasm, oral lichenoid reaction, oral mucosal roughening, lip erosion, salivary gland disorder, salivary gland mass, salivary gland calculus, tongue papillae hypertrophy, macroglossia, tongue fungal infection, circumoral oedema, oral mucosal exfoliation, leukoplakia oral, palatal oedema, lip exfoliation, and hypoesthesia teeth.

Despite meeting the initial inclusion criteria of the anatomo-physiological framework of the oral cavity, 20 oral AEs were reported fewer than 3 times in the PEI dataset and were therefore trimmed from Table 2.

### Cross-database analysis

Of the 60 oral AEs included in the primary analysis, 12 were significantly more reported in the PEI dataset (OR and 95% CI > 1), whereas 29 were more reported in the VAERS dataset (Table 2).

**Fig. 1:**
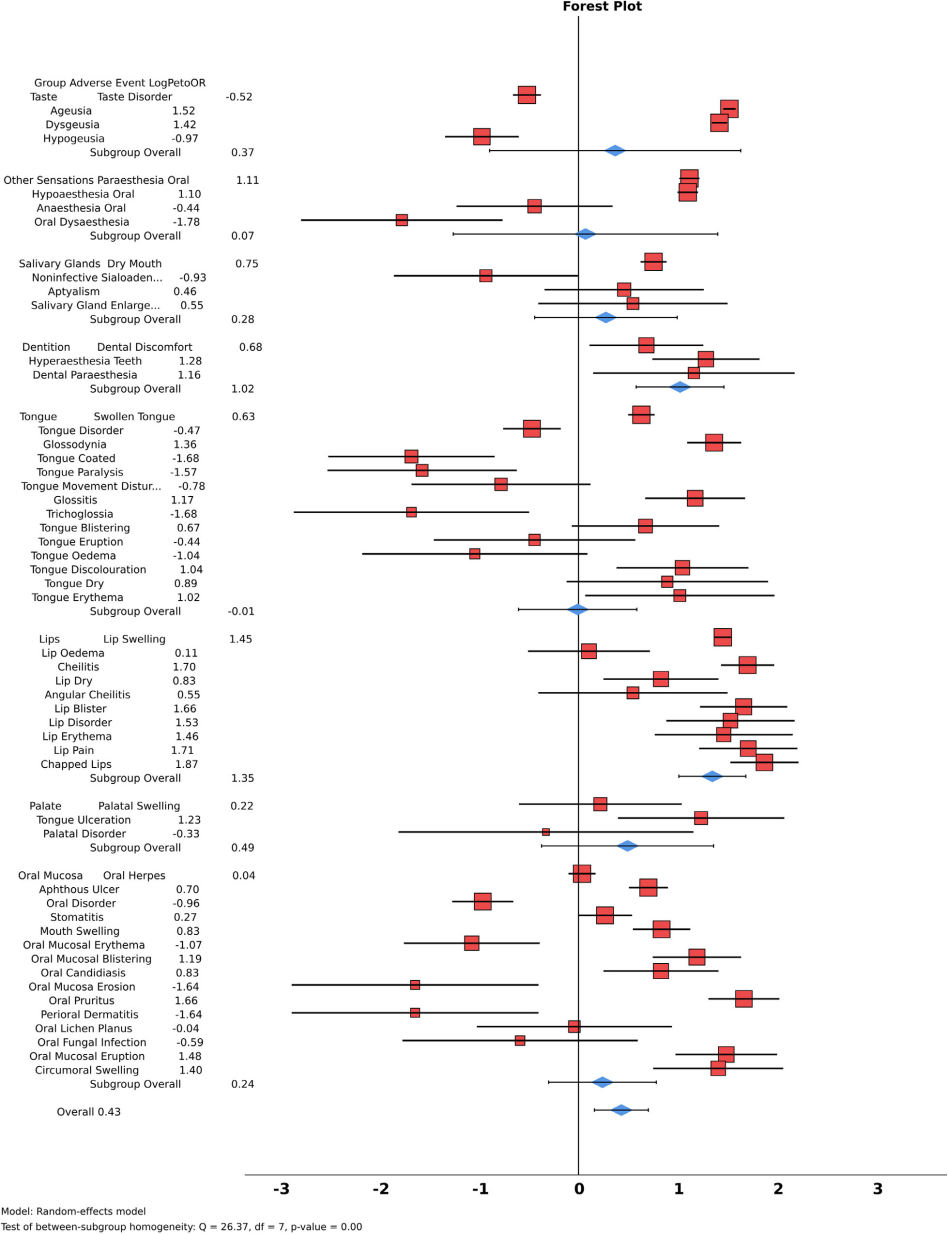
Cross-national comparison of oral adverse events (AEs) following COVID-19 vaccination in the PEI dataset (Germany, December 2020-December 2023) and the VAERS dataset (United States, December 2020-October 2024); PEI on right, VAERS on left. PEI, Paul-Ehrlich-Institut; VAERS, U.S. Vaccine Adverse Event Reporting System.

Compared to the VAERS dataset, taste disorder (OR: 1.69, 95% CI: [1.47-1.96]), hypogeusia ‘reduced taste’ (2.85 [1.88-4.33]), oral mucosal erythema (3.26 [1.47-7.22]), and broader, nonspecific terms such as oral disorder (2.82 [1.99-3.98]) and tongue disorder (1.61 [1.19-2.16]) were more frequently reported in the PEI dataset. On the other hand, ageusia (OR: 0.13 [0.12-0.14]), dysgeusia (0.16 [0.15-0.18]), oral paraesthesia (0.28 [0.25-0.32]), oral hypoaesthesia (0.29 [0.25-0.32]), swollen tongue (0.52 [0.45-0.60]), lip swelling (0.15 [0.13-0.17]), and mouth swelling (0.41 [0.30-0.56]) were more frequently reported in the VAERS dataset (Figure 1).

### Disproportionality analysis

Due to insufficient reports in the VAERS dataset, either for COVID-19 vaccines or for comparator vaccines, 11 oral AEs were disqualified from the disproportionality analysis. Among the remaining AEs, 21 met both the prespecified frequentist and Bayesian criteria and were classified as true SDRs, while 26 did not. In the VAERS dataset, taste disorder had the highest PRR (17.57 [10.28-30.03]), followed by ageusia (15.51 [13.39-17.97]), dental paraesthesia ‘altered sensation in teeth’ (9.97 [1.30-76.69]), hyperaesthesia teeth ‘teeth hypersensitivity’ (9.14 [3.28-25.44]), and oral anaesthesia (8.31 [1.06-64.91]). In addition, among the true SDRs, dysgeusia (3.31 [3.02-3.63]), oral paraesthesia (4.47 [3.87-5.16]), oral hypoaesthesia (4.26 [3.69-4.92]), swollen tongue (2.70 [2.28-3.19]), dry mouth (2.59 [2.21-3.03]), and aphthous ulcer (4.48 [3.32-6.04]) also exhibited significant disproportionality (Table 2).

### Subgroup analysis

**Fig. 2:**
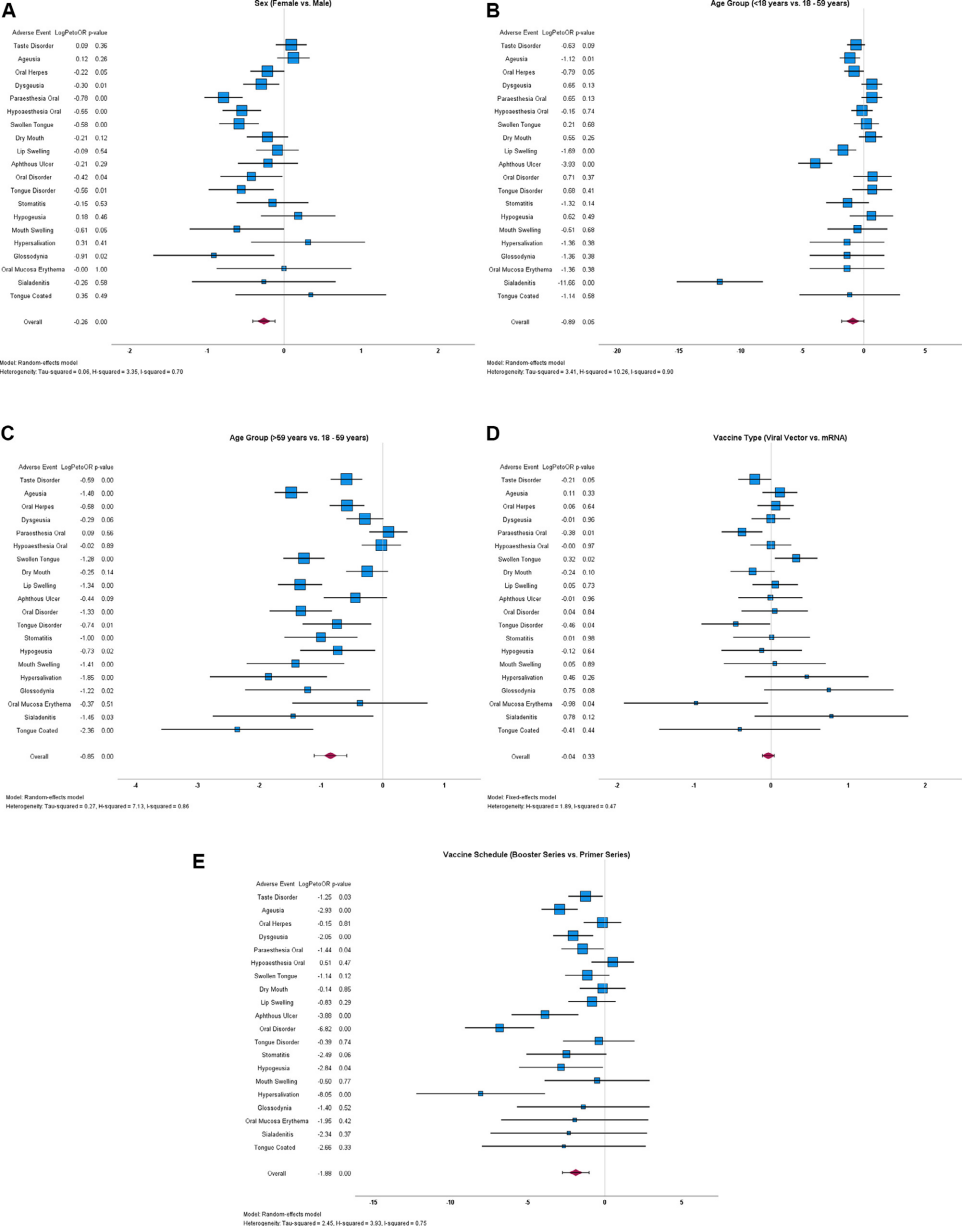
Forest plots of oral adverse events (AEs) following COVID-19 vaccination in the Paul-Ehrlich-Institute (PEI) dataset (Germany, December 2020-December 2023), stratified by (A) sex, (B and C) age groups, (D) vaccine platform, and (E) vaccination schedule sincerely.

Sex stratification that 6 of the top 20 oral AEs were significantly more reported by females, eg, oral paraesthesia (ARR 0.393 vs 0.141; *P* < .001), oral hypoaesthesia (0.366 vs 0.192; *P* < .001), and swollen tongue (0.348 vs 0.174; *P* < .001), while the remaining AEs showed no significant differences (Figure 2A).

Minors (<18 years) had six oral AEs that were significantly more reported than in adults (18-59 years), eg, taste disorder (0.706 vs 0.432; *P* < .001), ageusia (0.706 vs 0.328; *P* < .001), oral herpes (0.647 vs 0.359; *P* < .001), lip swelling (0.530 vs 0.193; *P* < .001), and aphthous ulcer (0.588 vs 0.111; *P* < .001). No significant differences were observed for the remaining AEs (Figure 2B).

Seniors (>59 years) had 14 oral AEs that were significantly more reported than in adults (18-59 years), eg, taste disorder (0.712 vs 0.432; *P* < .001), ageusia (0.965 vs 0.328; *P* < .001), swollen tongue (0.582 vs 0.224; *P* < 0.001), lip swelling (0.520 vs 0.193; *P* < .001), and stomatitis (0.164 vs 0.075; *P* < .001; Figure 2C).

In 2023, taste disorder (0.986; *P* < .001), ageusia (1.130; *P* < .001), and hypersalivation (0.126; *P* = .002) were significantly more reported, while swollen tongue (0.352; *P* = .001) was more frequently reported in 2021 compared to other years (Table 3).

**Table 3:** Stratification of top oral adverse events by sex, age group, and year in the Paul-Ehrlich-Institute (PEI) dataset (Germany, December 2020-December 2023).

Preferred term	Sex			Age group				Year			
	Female *N* (ARR)	Male *N* (ARR)	*P*	<18 y *N* (ARR)	18-59 y *N* (ARR)	>59 y *N* (ARR)	*P*	2021	2022	2023	*P*
Taste disorder	333 (0.479)	142 (0.525)	.390	12 (0.706)	334 (0.432)	104 (0.712)	**<.001**	263 (0.437)	163 (0.513)	55 (0.986)	**<.001**
Ageusia	299 (0.43)	131 (0.485)	.282	12 (0.706)	254 (0.328)	141 (0.965)	**<.001**	200 (0.332)	175 (0.551)	63 (1.130)	**<.001**
Oral herpes	300 (0.432)	93 (0.344)	.062	11 (0.647)	278 (0.359)	86 (0.588)	**<.001**	254 (0.422)	134 (0.422)	13 (0.233)	.130
Dysgeusia	272 (0.392)	77 (0.285)	**.016**	2 (0.118)	256 (0.331)	63 (0.431)	**.046**	250 (0.416)	79 (0.249)	23 (0.413)	**<.001**
Paraesthesia oral	273 (0.393)	38 (0.141)	**<.001**	2 (0.118)	256 (0.331)	44 (0.301)	.308	220 (0.366)	79 (0.249)	18 (0.323)	**.022**
Hypoaesthesia oral	254 (0.366)	52 (0.192)	**<.001**	6 (0.353)	238 (0.308)	46 (0.315)	.939	230 (0.382)	71 (0.224)	10 (0.179)	**<.001**
Swollen tongue	242 (0.348)	47 (0.174)	**<.001**	3 (0.177)	173 (0.224)	85 (0.582)	**<.001**	212 (0.352)	64 (0.202)	14 (0.251)	**.001**
Dry mouth	203 (0.292)	63 (0.233)	.132	2 (0.118)	201 (0.260)	48 (0.328)	.177	178 (0.296)	68 (0.214)	24 (0.430)	**.013**
Lip swelling	185 (0.266)	66 (0.244)	.591	9 (0.530)	149 (0.193)	76 (0.520)	**<.001**	173 (0.288)	68 (0.214)	11 (0.197)	.109
Aphthous ulcer	96 (0.138)	30 (0.111)	.341	10 (0.588)	86 (0.111)	24 (0.164)	**<.001**	63 (0.105)	55 (0.173)	8 (0.143)	**.037**
Oral disorder	95 (0.137)	23 (0.085)	.050	0 (0)	75 (0.097)	38 (0.260)	**<.001**	66 (0.110)	38 (0.120)	15 (0.269)	**.024**
Tongue disorder	89 (0.128)	18 (0.067)	**.013**	0 (0)	69 (0.089)	24 (0.164)	**.021**	65 (0.108)	32 (0.101)	14 (0.251)	**.029**
Stomatitis	66 (0.095)	22 (0.081)	.609	3 (0.177)	58 (0.075)	24 (0.164)	**.003**	49 (0.081)	34 (0.107)	5 (0.09)	.471
Hypogeusia	56 (0.081)	26 (0.096)	.535	0 (0)	58 (0.075)	20 (0.137)	**.044**	42 (0.07)	29 (0.091)	11 (0.197)	**.020**
Mouth swelling	43 (0.062)	8 (0.030)	.071	1 (0.059)	30 (0.039)	16 (0.109)	**.004**	40 (0.066)	11 (0.035)	1 (0.018)	.086
Hyper salivation	23 (0.033)	12 (0.044)	.524	1 (0.059)	19 (0.025)	13 (0.089)	**.001**	13 (0.022)	15 (0.047)	7 (0.126)	**.002**
Glossodynia	28 (0.040)	3 (0.011)	**.026**	1 (0.059)	19 (0.025)	9 (0.062)	**.045**	16 (0.027)	14 (0.044)	1 (0.018)	.344
Oral mucosal erythema	18 (0.026)	7 (0.026)	1.000	1 (0.059)	19 (0.025)	5 (0.034)	.347	18 (0.03)	5 (0.016)	2 (0.036)	.313
Sialadenitis	17 (0.024)	5 (0.018)	.753	4 (0.235)	11 (0.014)	6 (0.041)	**<.001**	8 (0.013)	13 (0.041)	1 (0.018)	**.029**
Tongue coated	13 (0.019)	7 (0.026)	.655	0 (0)	10 (0.013)	9 (0.062)	**.003**	14 (0.023)	3 (0.009)	3 (0.054)	.073

Boldface indicates statistical significance (p<0.05).

The absolute reporting ratio (ARR) is measured per each 1000 individual case safety reports.

Chi-squared (χ^2^) test and Fisher’s exact test were used with a significance level of *P* < 0.05.

No oral AEs were reported following inactivated virus vaccines, while only 8 were reported after protein subunit vaccines. All top 20 oral AEs were reported following mRNA and viral vector vaccines (Table 4).

**Table 4:** Stratification of top oral adverse events by vaccine type and schedule in the Paul-Ehrlich-Institute (PEI) dataset (Germany, December 2020-December 2023).

Preferred term	Vaccine type					Vaccine schedule		
	mRNA *N* (ARR)	Viral vector *N* (ARR)	Protein subunit *N* (ARR)	Inactivated virus *N* (ARR)	*P*	Primer series *N* (ARR)	Booster series *N* (ARR)	*P*
Taste disorder	348 (0.467)	126 (0.572)	3 (0.820)	0 (0)	.081	470 (0.488)	7 (1.104)	.055
Ageusia	338 (0.454)	89 (0.404)	3 (0.820)	0 (0)	.252	419 (0.435)	11 (1.735)	**<.001**
Oral herpes	308 (0.413)	86 (0.390)	4 (1.093)	0 (0)	.116	395 (0.41)	3 (0.473)	.749
Dysgeusia	269 (0.361)	80 (0.363)	3 (0.820)	0 (0)	.278	345 (0.358)	7 (1.104)	**.006**
Paraesthesia oral	220 (0.295)	92 (0.417)	2 (0.547)	0 (0)	**.017**	309 (0.321)	5 (0.789)	.087
Hypoaesthesia oral	239 (0.321)	71 (0.322)	1 (0.273)	0 (0)	.981	310 (0.322)	1 (0.158)	.728
Swollen tongue	237 (0.318)	49 (0.222)	1 (0.273)	0 (0)	.064	283 (0.294)	4 (0.631)	.121
Dry mouth	194 (0.26)	72 (0.327)	2 (0.547)	0 (0)	.106	266 (0.276)	2 (0.315)	.696
Lip swelling	196 (0.263)	55 (0.250)	0 (0)	0 (0)	.909	248 (0.258)	3 (0.473)	.227
Aphthous ulcer	97 (0.13)	29 (0.132)	0 (0)	0 (0)	.948	122 (0.127)	4 (0.631)	**.010**
Oral disorder	92 (0.123)	26 (0.118)	0 (0)	0 (0)	.944	112 (0.116)	6 (0.946)	**<.001**
Tongue disorder	76 (0.102)	34 (0.154)	0 (0)	0 (0)	.140	109 (0.113)	1 (0.158)	.514
Stomatitis	68 (0.091)	20 (0.091)	0 (0)	0 (0)	1.000	86 (0.089)	2 (0.315)	.113
Hypogeusia	60 (0.081)	20 (0.091)	0 (0)	0 (0)	.771	78 (0.081)	2 (0.315)	.097
Mouth swelling	39 (0.052)	11 (0.050)	0 (0)	0 (0)	1.000	50 (0.052)	0 (0)	1.000
Hyper salivation	29 (0.039)	5 (0.023)	0 (0)	0 (0)	.395	32 (0.033)	2 (0.315)	**.021**
Glossodynia	28 (0.038)	3 (0.014)	0 (0)	0 (0)	.190	31 (0.032)	0 (0)	1.000
Oral mucosal erythema	15 (0.020)	10 (0.045)	0 (0)	0 (0)	.140	25 (0.026)	0 (0)	1.000
Sialadenitis	20 (0.027)	2 (0.009)	0 (0)	0 (0)	.264	22 (0.023)	0 (0)	1.000
Tongue coated	14 (0.019)	6 (0.027)	0 (0)	0 (0)	.471	20 (0.021)	0 (0)	1.000

Boldface indicates statistical significance (p<0.05).

The absolute reporting ratio (ARR) is measured per each 1000 individual case safety reports.

Chi-squared (χ^2^) test and Fisher’s exact test were used with a significance level of *P* < .05.

Compared to mRNA vaccines, viral vector vaccines had a significantly higher reporting rate of oral paraesthesia (0.417 vs 0.295; *P* = .006). Conversely, swollen tongue was more frequently reported following mRNA vaccines (0.318 vs 0.222; *P* = .026; Figure 2D).

The booster series had a significantly higher reporting rate for 5 oral AEs, including ageusia (1.735 vs 0.435; *P* < .001), dysgeusia (1.104 vs 0.358; *P* = .006), aphthous ulcer (0.631 vs 0.127; *P* = .010), oral disorder (0.946 vs 0.116; *P* < .001), and hypersalivation (0.315 vs 0.033; *P* = .021). No oral AE was significantly more reported following the primer series, and 4 of the top 20 oral AEs were not reported at all after the booster series (Figure 2E).

### Regression analysis

The following statistically significant associations were derived from multivariable logistic regression models, where each oral AE was treated as the dependent variable, and sex, age group, vaccine type, and vaccination schedule were entered as independent predictors.

Females had significantly higher adjusted odds ratio (aOR) of reporting 5 oral AEs, ie, oral herpes (aOR: 1.39, 95% CI: [1.09-1.77]), dysgeusia (1.34 [1.03-1.74]), oral paraesthesia (2.71 [1.92-3.83]), oral hypoaesthesia (2.04 [1.49-2.80]), swollen tongue (2.08 [1.50-2.89]), oral disorder (1.66 [1.04-2.63]), tongue disorder (1.91 [1.11-3.28]), and glossodynia (3.68 [1.11-12.23]) (Table 5).

**Table 5:** Multivariable logistic regression of top oral adverse events following COVID-19 vaccination in the Paul-Ehrlich-Institute (PEI) dataset (Germany; December 2020-December 2023).

Preferred Term	Sex	Age group		Vaccine type		Vaccine schedule
	Female vs Male aOR (95% CI)	< 18 vs 18-59 y aOR (95% CI)	> 59 vs 18-59 y aOR (95% CI)	Vector vs mRNA aOR (95% CI)	Prot. Sub. vs mRNA aOR (95% CI)	Booster vs Primer aOR (95% CI)
Taste disorder	0.99 (0.81-1.22)	1.76 (0.99-3.15)	**1.64 (1.31**-**2.05)**	1.22 (0.99-1.51)	1.97 (0.63-6.16)	**2.19 (1.03**-**4.65)**
Ageusia	0.99 (0.80-1.22)	**2.04 (1.11**-**3.75)**	**2.99 (2.42**-**3.70)**	0.87 (0.68-1.12)	2.20 (0.70-6.86)	**3.00 (1.64**-**5.51)**
Oral herpes	**1.39 (1.09**-**1.77)**	**1.95 (1.06**-**3.58)**	**1.73 (1.36**-**2.22)**	0.95 (0.74-1.22)	**2.96 (1.10**-**7.94)**	1.08 (0.35-3.39)
Dysgeusia	**1.34 (1.03**-**1.74)**	0.39 (0.10-1.57)	1.31 (0.99-1.73)	1.06 (0.82-1.38)	2.55 (0.82-7.97)	**3.20 (1.50**-**6.83)**
Paraesthesia oral	**2.71 (1.92**-**3.83)**	0.48 (0.12-1.92)	0.97 (0.70-1.35)	**1.54 (1.20**-**1.98)**	1.98 (0.49-7.99)	**3.16 (1.30**-**7.72)**
Hypoaesthesia oral	**2.04 (1.49**-**2.80)**	1.31 (0.58-2.95)	1.10 (0.80-1.52)	1.02 (0.77-1.35)	0.91 (0.13-6.52)	0.55 (0.08-3.90)
Swollen tongue	**2.08 (1.50**-**2.89)**	0.85 (0.27-2.67)	**2.87 (2.21**-**3.74)**	**0.72 (0.53**-**0.99)**	1.00 (0.14-7.16)	1.70 (0.63-4.59)
Dry mouth	1.29 (0.96-1.73)	0.51 (0.13-2.07)	1.26 (0.91-1.73)	1.29 (0.98-1.71)	2.28 (0.57-9.19)	1.24 (0.31-5.00)
Lip swelling	1.20 (0.90-1.61)	**2.89 (1.47**-**5.69)**	**2.78 (2.10**-**3.68)**	1.02 (0.75-1.39)	*NA*	1.48 (0.47-4.67)
Aphthous ulcer	1.34 (0.88-2.04)	**5.86 (3.01**-**11.40)**	1.48 (0.94-2.33)	1.18 (0.77-1.80)	*NA*	**3.91 (1.22**-**12.49)**
Oral disorder	**1.66 (1.04**-**2.63)**	*NA*	**2.63 (1.76**-**3.94)**	1.03 (0.66-1.61)	*NA*	**6.61 (2.85**-**15.35)**
Tongue disorder	**1.91 (1.11**-**3.28)**	*NA*	**1.90 (1.18**-**3.06)**	1.55 (0.99-2.42)	*NA*	1.64 (0.23-11.88)
Stomatitis	1.39 (0.84-2.30)	*NA*	**2.23 (1.38**-**3.61)**	1.05 (0.63-1.73)	*NA*	3.00 (0.73-12.38)
Hypogeusia	0.78 (0.49-1.26)	*NA*	**1.80 (1.07**-**3.02)**	1.08 (0.64-1.82)	*NA*	3.25 (0.79-13.47)
Mouth swelling	1.98 (0.92-4.27)	1.89 (0.26-14.03)	**3.42 (1.84**-**6.37)**	1.06 (0.54-2.11)	*NA*	*NA*
Hyper salivation	0.87 (0.42-1.81)	2.35 (0.31-17.78)	**3.60 (1.74**-**7.46)**	0.63 (0.24-1.65)	*NA*	**6.03 (1.40**-**26.07)**
Glossodynia	**3.68 (1.11**-**12.23)**	2.40 (0.32-18.03)	**3.00 (1.35**-**6.66)**	0.24 (0.06-1.02)	*NA*	*NA*
Oral mucosal erythema	1.07 (0.44-2.57)	3.03 (0.40-23.19)	1.37 (0.51-3.70)	**2.26 (1.01**-**5.09)**	*NA*	*NA*
Sialadenitis	1.53 (0.55-4.21)	**15.55 (4.86**-**49.77)**	**3.19 (1.17**-**8.69)**	0.40 (0.09-1.76)	*NA*	*NA*
Tongue coated	0.78 (0.30-1.99)	*NA*	**4.66 (1.88-11.57)**	1.37 (0.52-3.61)	*NA*	*NA*

Adjusted odds ratios (aOR) that are statistically significant (*P* < .05) are in bold.

Minors had significantly higher odds of reporting ageusia (2.04 [1.11-3.75]), oral herpes (1.95 [1.06-3.58]), lip swelling (2.89 [1.47-5.69]), aphthous ulcer (5.86 [3.01-11.40]), and sialadenitis (15.55 [4.86-49.77]). Seniors had significantly higher odds of reporting 14 oral AEs, eg, taste disorder (1.64 [1.31-2.05]), ageusia (2.99 [2.42-3.70]), and hypogeusia (1.80 [1.07-3.02]) (Table 5).

Viral vector vaccines were associated with higher odds of reporting oral paraesthesia (1.54 [1.20-1.98]) and oral mucosal erythema (2.26 [1.01-5.09]) but lower odds of swollen tongue (0.72 [0.53-0.99]). Moreover, booster series had significantly higher odds of reporting taste disorder (2.19 [1.03-4.65]), ageusia (3.00 [1.64-5.51]), dysgeusia (3.20 [1.50-6.83]), oral paraesthesia (3.16 [1.30-7.72]), and hypersalivation (6.03 [1.40-26.07]) (Table 5).

## Discussion

This study provided a comprehensive pharmacovigilance assessment of oral AEs following COVID-19 vaccination in Germany, revealing that sensory disturbances, eg, taste disorder, ageusia, dysgeusia, oral paraesthesia, and oral hypoaesthesia, were the most frequently reported AEs. Cross-database comparisons further confirmed an increased reporting pattern of these sensory AEs in the United States, reinforcing their relevance in postvaccination safety monitoring. Disproportionality analysis provided an additional layer of validation through classifying these AEs as true SDRs for COVID-19 vaccines. Moreover, cross-database analyses identified differences in reporting trends between Germany and the United States, likely influenced by structural factors rather than merely biodemographic variations. Females exhibited significantly higher reporting rates for several oral AEs, while minors and seniors also displayed distinct susceptibility patterns as compared with middle-aged adults (18-59 years). These findings emphasise the need for continued pharmacovigilance to better characterise the clinical relevance of oral AEs associated with COVID-19 vaccination.

### Oral sensory AEs

Gustatory AEs, including taste disorders, ageusia, and dysgeusia, were the most frequently reported oral AEs in German national pharmacovigilance data, a pattern also observed in datasets from the United States, the European Union, and Australia.^6^, ^7^, ^8^ In EudraVigilance, dysgeusia, ageusia, and taste disorder ranked among the top 10 oral AEs, with ARRs of 3.81, 2.96, and 1.73 cases per 1000 ICSRs, respectively.^7^ Similarly, in the Australian Database of Adverse Event Notifications (DAEN), dysgeusia (7.40), taste disorder (2.73), and ageusia (2.59) were also among the top 10 oral AEs.^8^ COVID-19 vaccines exhibited significantly higher ARRs of gustatory AEs in VAERS compared to seasonal influenza vaccines.^6^

One of the proposed hypotheses for this increased reporting of gustatory AEs is notoriety bias which can be defined as the increased reporting of AEs when a medical intervention, such as a vaccine or drug, gains heightened public attention, media coverage, or regulatory scrutiny, leading to a disproportionate perception of its risk.^14^ Supporting this hypothesis, the VAERS database showed a sharp rise in gustatory AEs during the COVID-19 pandemic, with a significant increase in reporting after 2020 compared to the prepandemic interval (eg, ageusia: 0.062 vs 0.003; *P* < .001; taste disorder: 0.028 vs 0.001; *P* < .001).^6^

Being labelled as characteristic symptoms of SARS-CoV-2 infection, olfactory and gustatory dysfunctions attracted significant public attention; therefore, they were enlisted among the solicited adverse reactions to COVID-19 vaccines in numerous postmarketing active surveillance studies, such as Network of Data Sources for Vaccine Safety Evaluation (SAFETY-VAC) and COVID Vaccine Monitor.^17^, ^18^, ^19^ In 2023, the Brighton Collaboration, a global vaccine safety research network dedicated to advancing pharmacovigilance science, published a case definition of anosmia ‘loss of smell’ along with guidelines for its analysis in immunisation safety data in response to the growing interest in chemosensory dysfunction reports.^20^ Moreover, the EMA-funded VACcine COVID-19 monitoring readinESS project developed a case definition for anosmia and ageusia, incorporating background rates to support postauthorisation surveillance and signal detection efforts.^19^

Bahri et al^21^ proposed several alternative hypotheses to explain postvaccination anosmia and ageusia instead of attributing them to the nocebo effect. One hypothesis suggests that partial demyelination of taste-related neural pathways may impair gustatory function, similar to its proposed role in anosmia.^22^ Another hypothesis attributes gustatory dysfunction to vaccine-induced inflammation in the oral neuroepithelium, leading to transient sensory impairment. In addition, direct interaction between vaccine-induced spike proteins and taste receptors or immune-mediated damage to gustatory cells has been suggested as a potential mechanism.^23^

Other sensory AEs, such as oral paraesthesia and oral hypoaesthesia, were also among the most frequently reported oral AEs in the German dataset, similar to findings in the Australian DAEN (7.53 and 1.56, respectively), EudraVigilance (3.15 and 2.10, respectively), and VAERS (8.72 and 6.48, respectively).^8^ On October 6, 2021, the Pharmacovigilance Risk Assessment Committee of the EMA issued an updated safety guidance for the Comirnaty vaccine, recommending the inclusion of paraesthesia (an unusual skin sensation such as tingling or crawling) and hypoesthesia (reduced skin sensitivity) as recognised side effects in the product information.^24^ A postmarketing-active surveillance study of mRNA-based vaccines identified oral paraesthesia as the most frequently reported oral AE, with an overall frequency of 1.3%. Notably, this AE was not among the solicited options for participants, suggesting that the true prevalence may be underestimated.^25^

### Oral mucosal AEs

Oral herpes was among the most frequently reported oral AEs in the German database (ARR, 0.41), consistent with findings from the Australian DAEN (1.27) and VAERS (1.89).^8^ Several case reports have described oral herpes zoster following COVID-19 vaccination, though their aetiologic evidence remains inconclusive.^26^^,^^27^ To further investigate this, Akpandak et al^28^ conducted a self-controlled case-series analysis in the VAERS database, assessing the risk of herpes zoster reactivation post-COVID-19 vaccination. Their findings showed an incidence rate ratio of 0.91, suggesting no increased risk compared to influenza vaccination during the prepandemic and early pandemic periods. Contrarily, a retrospective cohort study from Turkey reported a significant increase in herpes zoster cases in 2021, coinciding with the introduction of COVID-19 vaccination, suggesting a potential association between vaccination and herpes zoster reactivation, particularly in recipients of inactivated vaccines.^29^

Aphthous ulcers emerged as another common oral mucosal AE in Germany (0.13), the United States (0.27), and Australia (0.28).^8^ An earlier review of VAERS identified 368 cases of oral aphthous ulcers compared to 126 cases of genital and vulvovaginal ulcers following COVID-19 vaccination, thus indicating the susceptibility of the oral mucosa to postvaccination ulcerations.^30^ Consistently, multiple case reports have described aphthous stomatitis following COVID-19 vaccination, though its aetiology remains unclear.^31^^,^^32^ A recent familial case report contextualised aphthous stomatitis within PFAPA syndrome (periodic fever, aphthous stomatitis, pharyngitis, and cervical adenitis), attributing it to a TNFAIP3 mutation with potential modulation by environmental factors, including COVID-19 vaccination.^33^

Swollen tongue, lip swelling, and mouth swelling were frequently reported oral AEs in Germany (0.30, 0.26, and 0.05, respectively), the United States (0.58, 1.69, and 0.131, respectively), and Australia (5.16, 4.94, and 0.53, respectively).^8^ While lip swelling is a diagnostic criterion for anaphylaxis, the Brighton Collaboration has refined its classification to distinguish between cases associated with respiratory distress – indicative of anaphylaxis – and those presenting as isolated angioedema, improving specificity in vaccine safety surveillance.^34^ Likewise, tongue swelling continues to serve as a key marker of severe hypersensitivity reactions, particularly when linked to airway compromise, warranting increased vigilance in postvaccination monitoring.^34^ Future research on oral AEs should employ the self-controlled case-series methodology to enhance understanding of oral mucosal and anaphylactic AEs and improve signal validation.^35^

### Cross-database comparison of oral AEs

The decision to incorporate cross-database analysis in this study is justified not only by the absence of comparator vaccines or drugs for contextualising oral AEs following COVID-19 vaccination but also by the broader advantages of multidatabase pharmacovigilance.^36^ Trifirò et al^36^ highlighted that integrating multiple healthcare databases enhances statistical power, improves signal detection, and accounts for variations in reporting behaviours across regions, ultimately strengthening postmarketing vaccine safety assessment​.

Bonaldo et al^37^ analysed rotavirus vaccine safety using VAERS and VigiBase of the World Health Organisation, finding that VAERS predominantly reported RotaTeq cases (86.2%), while VigiBase had more reports for Rotarix (67.7%). VAERS had a higher overall report volume, likely due to U.S. reporting practices, whereas VigiBase captured a broader global perspective with more diverse AE distributions. Thus, it highlighted that cross-database differences should not be directly attributed to biodemographic variability but rather to variations in reporting systems and pharmacovigilance practices.

For COVID-19 vaccines, cross-database analyses have been widely utilised; for example, acute nephrotoxic AEs were reported disproportionately more often in VAERS compared to EudraVigilance.^38^ Montano^39^ analysed serious AEs, finding that EudraVigilance had a stronger association between Moderna and serious AEs, and VAERS showed no major differences between manufacturers, with overall risk estimates consistently higher in EudraVigilance than in VAERS.

Oral SDRs

According to the EMA, the safety signal management process consists of 4 steps^1^: signal detection, which utilises disproportionality analysis or clinical judgement (if conducted manually) and typically generates SDRs^2^; signal validation, which involves clinical assessment to evaluate the novelty and strength of evidence, determining whether a signal is validated or not^3^; signal assessment, where validated signals undergo comprehensive review using all available data, from preclinical to postmarketing, potentially leading to the classification of a potential or identified risk; and^4^ regulatory action and risk communication.^40^ Given this structured approach, SDRs should be viewed as an initial step in signal confirmation rather than definitive evidence of risk.^14^ There is increasingly compelling evidence supporting the use of a hybrid approach for SDR identification by integrating predefined frequentist and Bayesian conditions; therefore, both methods were applied in this study.^13^ In the context of oral AEs, even in the most extreme scenario where an oral AE – whether sensory or mucosal – is validated and confirmed, it is likely to result only in its inclusion in the product information rather than any regulatory action restricting vaccine dissemination. Notably, our disproportionality analysis identified 21 oral AEs as true SDRs, most of which involved sensory disturbances and mucosal symptoms. While these SDRs do not imply causality, their recurrence across datasets and significant signal strength merit further clinical evaluation and consideration for inclusion in vaccine product labelling, particularly where patient reassurance and informed consent are concerned.

### Sex- and age-based reporting disparities of oral AEs

In Germany, females accounted for nearly 72% of all pharmacovigilance reports related to COVID-19 vaccines, whereas males contributed approximately 28%, despite both sexes exhibiting similar vaccination uptake rates (basic immunisation with 2 primer doses: 13% female vs 14% male; 1 booster: 66% vs 66%; 2 boosters: 14% vs 12%; zero doses: 8% vs 8%).^11^^,^^41^ This sex-based disparity in AE reporting may stem from a combination of biological and psychosocial factors. A Dutch cohort study on COVID-19 vaccine safety by Duijster et al^42^ found that females had nearly twice the odds of reporting AEs compared to males, with the greatest differences observed after the first dose. Proposed explanations include stronger humoral and cell-mediated immune responses, driven by oestrogen-enhanced immune activation and testosterone-induced immunosuppression, as well as genetic factors, given that the X chromosome carries more immune-related genes than the Y chromosome.^42^^,^^43^ Beyond biological mechanisms, higher baseline symptom prevalence (eg, headache, nausea), greater health awareness, and an increased tendency to report symptoms may further contribute to this disparity.^42^

In the present study of oral AEs in German pharmacovigilance data, univariate analysis indicated that sex-based differences in reporting were observed only for certain oral AEs, such as oral paraesthesia and oral hypoaesthesia, with all statistically significant differences skewed towards females. This pattern was further confirmed by multivariable logistic regression, which identified additional oral AEs reported more frequently by females than males, with no single oral AE being more commonly reported by males. Prior analyses of oral AEs in U.S. VAERS, EudraVigilance, and Australian DAEN consistently observed this sex-based reporting disparity.^6^, ^7^, ^8^

Age stratification of German pharmacovigilance data revealed that minors (<18 years) and seniors (>59 years) exhibited a higher reporting tendency for oral AEs compared to middle-aged adults (18-59 years). This pattern contrasts with findings from the Australian DAEN and EudraVigilance databases, where these two age brackets had lower reporting rates.^7^^,^^8^ While the reasons for this age-based disparity remain unclear, drawing conclusions about age-related susceptibility to oral AEs based solely on reporting trends may be misleading and should not be used to question vaccine safety. A more granular assessment at the AE level, along with a case-by-case review, is required before extrapolating age-related disparities.^14^

### COVID-19 vaccine type and schedule

Differences in oral AEs between mRNA-based and viral vector vaccines were limited to a few specific events and lacked a consistent pattern, making it impossible to attribute higher reporting trends to either vaccine type. A similar observation was previously reported in the Australian DAEN and U.S. VAERS databases, where stratification by vaccine type revealed no clear direction in reporting disparities.^6^^,^^8^ While a few exceptions reached statistical significance within each vaccine type, their verification requires a more granular, case-by-case analysis beyond the scope of whole-database assessments.

The higher odds of oral AEs reporting following booster series compared to primer series should be interpreted with caution, as the available sample size for booster series did not exceed 0.65% of the German dataset.^11^ This small sample size increases variability and may introduce instability in the estimates, akin to the uncertainty seen in the right tail of Kaplan–Meier curves due to late-stage attrition.^44^ One possible explanation for this finding is the reductive nature of the German pharmacovigilance dataset, where each ICSR is assigned a single clinical symptom labelled as the ‘chief complaint’.^11^ Given that oral AEs remain unsolicited in product information, vaccine recipients may be more inclined to report them during the booster stage as unexpected reactions, whereas common solicited AEs such as headache and fever may be deprioritised in spontaneous reporting, thereby inflating the relative proportion of oral AEs in the booster subgroup.

### Strengths

This study has several methodological strengths that enhance the validity of its findings on oral AEs. The cross-database comparison between German PEI and U.S. VAERS data provides contextual validation of reporting patterns across distinct pharmacovigilance systems. The hybrid statistical approach of disproportionality analysis, combining frequentist and Bayesian methods, improves signal specificity and minimises false positives. In addition, multivariable regression strengthens the analysis by controlling for demographic and vaccine-related confounders, reducing the risk of spurious associations from univariate analyses. Notably, this study is the first to examine Germany's national pharmacovigilance data on oral AEs, contributing to a broader understanding of postvaccination safety monitoring.

### Limitations

The study is also subject to the inherent limitations of passive surveillance systems, including reporting biases, underreporting, and inconsistent data quality. In addition, the absence of comparator data in the German PEI dataset prevented a disproportionality analysis of oral AEs within the German data alone, necessitating the use of cross-database analysis for contextual validation. Structural differences in AE annotation between U.S. VAERS and German PEI further complicated direct comparisons, as VAERS assigns up to 5 medical dictionary for regulatory activities-preferred terms per ICSR, whereas PEI attributes only a single term per report. However, the shared use of medical dictionary for regulatory activities terminology facilitated signal classification, with the primary symptom in VAERS serving as an approximation of the chief complaint in PEI data. Moreover, the reductive classification approach of PEI may have led to an underestimation of oral AE reporting rates, as these events often present alongside systemic reactions rather than in isolation. For example, gustatory AEs frequently co-occur with olfactory disturbances, which may not have been captured comprehensively in the German dataset.

### Implications

In line with the EMA Good Vigilance Practice Module IX framework, future research should progress from the signal detection phase to the signal validation phase for oral AEs identified as SDRs. Self-controlled case-series designs are recommended for clinically significant mucosal AEs, such as oral herpes, aphthous stomatitis, and oral lichen planus, to examine potential causality. In addition, a granular assessment is needed to verify age and vaccine type–based disparities in oral AE reporting. From a regulatory perspective, while no severe actions appear necessary, validated oral AEs, particularly sensory disturbances, may warrant inclusion in vaccine product information to improve transparency and public awareness of potential postvaccination effects. Dental professionals should also be aware of these symptoms and their typically transient nature to provide appropriate reassurance and guidance to patients experiencing postvaccination oral AEs.

## Author contributions

Conceptualisation, Abanoub Riad; Methodology, Abanoub Riad; Formal analysis, Abanoub Riad; Writing – original draft preparation, Abanoub Riad; Supervision, Abanoub Riad; Funding acquisition, Abanoub Riad. The author approved the submitted version of the article.

## Ethics statement

This study used publicly available, deidentified data from PEI and VAERS; therefore, it did not require formal approval from an institutional ethics board or informed consent.

## Declaration of generative AI and AI-assisted technologies in the writing process

The author declares that Generative AI was used in the preparation of this manuscript. Specifically, OpenAI’s ChatGPT (version 4o) was utilised for proofreading and language refinement. This AI tool was employed solely to correct grammatical errors and enhance readability, with all substantive content independently developed by the author.

## Funding

This study was supported by the NPO ‘Systemic Risk Institute’ no. LX22NPO5101, funded by European Union–Next Generation EU (Ministry of Education, Youth and Sports, NPO: EXCELES).

## Conflict of interest

The author declares that the research was conducted without any commercial or financial relationships that could be construed as a potential conflict of interest.

Data availability statement

Publicly available datasets were analysed in this study. These data can be found at:

• https://www.pei.de/SharedDocs/Downloads/EN/newsroom-en/dossiers/reports-uaw/download-uaw-data-xls-2020-12-27-2023-12-31.html

• https://vaers.hhs.gov/data/datasets.html

## References

[1] Oster M.E., Shay D.K., Su J.R. (2022). Myocarditis cases reported after mRNA-based COVID-19 vaccination in the US from December 2020 to August 2021. JAMA.

[2] Jaiswal V., Mukherjee D., Peng Ang S. (2023). COVID-19 vaccine-associated myocarditis: analysis of the suspected cases reported to the EudraVigilance and a systematic review of the published literature. Int J Cardiol Heart Vasc.

[3] Shimabukuro T.T., Cole M., Su JR. (2021). Reports of anaphylaxis after receipt of mRNA COVID-19 vaccines in the US – December 14, 2020-January 18, 2021. JAMA.

[4] Tsuchiya H., Mizogami M. (2024). Characteristics of oral adverse effects following COVID-19 vaccination and similarities with oral symptoms in COVID-19 patients: taste and saliva secretory disorders. Med Princ Pract.

[5] Khazeei Tabari M.A., Najary S., Khadivi G., Yousefi M.J., Samieefar N., Abdollahimajd F. (2022). Oral lesions after COVID-19 vaccination: immune mechanisms and clinical approach. Infect Med.

[6] Riad A., Põld A., Kateeb E., Attia S. (2022). Oral adverse events following COVID-19 vaccination: analysis of VAERS reports. Front Public Health.

[7] Riad A., Schulz-Weidner N., Dziedzic A., Howaldt H.P., Attia S. (2023). Oral side effects of COVID-19 vaccines in 32 European countries: analysis of EudraVigilance reports. J Med Virol.

[8] Riad A., Issa J., Attia S., Dušek L., Klugar M. (2023). Oral adverse events following COVID-19 and influenza vaccination in Australia. Hum Vaccin Immunother.

[9] Fusaroli M., Salvo F., Begaud B. (2024). The REporting of A Disproportionality Analysis for DrUg Safety Signal Detection Using Individual Case Safety Reports in PharmacoVigilance (READUS-PV): explanation and elaboration. Drug Saf.

[10] Paul-Ehrlich-Institute (PEI) (2024). Safety of COVID-19 Vaccines.

[11] Paul-Ehrlich-Institut (PEI) (2024). Reports submitted to the Paul-Ehrlich-Institut of suspected side effects after the use of COVID-19 vaccines.

[12] European Medicines Agency. Guideline on good pharmacovigilance practices (GVP) – Module IX Addendum I – methodological aspects of signal detection from spontaneous reports of suspected adverse reactions. 2017 [Accessed 14 February 2025]. Available from: https://www.ema.europa.eu/en/documents/scientific-guideline/guideline-good-pharmacovigilance-practices-gvp-module-ix-addendum-i-methodological-aspects-signal-detection-spontaneous-reports-suspected-adverse-reactions_en.pdf

[13] Park G., Jung H., Heo S.J., Jung I. (2020). Comparison of data mining methods for the signal detection of adverse drug events with a hierarchical structure in postmarketing surveillance. Life.

[14] Cutroneo P.M., Sartori D., Tuccori M. (2024). Conducting and interpreting disproportionality analyses derived from spontaneous reporting systems. Front Drug Saf Regul.

[15] Bundesministerium der Justiz (BMJ) (2001). https://www.gesetze-im-internet.de/ifsg/.

[16] US-DHHS. VAERS data use guide. Vaccine Adverse Event Reporting System (VAERS). [Accessed 28 January 2025]. Available from: https://www.meddra.org/.

[17] Lee Y., Min P., Lee S., Kim S.W. (2020). Prevalence and duration of acute loss of smell or taste in COVID-19 patients. J Korean Med Sci.

[18] VAC4EU. EMA-funded studies. [Accessed 14 February 2025]. Available from: https://vac4eu.org/ema-funded-studies/

[19] Egbers T, Willame C, Belbachir L, et al. ACCESS-background rate of adverse events-definition –anosmia & ageusia [Accessed 10 March 2025]. Available from: https://zenodo.org/records/5236687

[20] Liu Y.C.C., Munoz F.M., Izurieta H.S. (2023). Anosmia: Brighton Collaboration case definition and guidelines for data collection, analysis, and presentation of immunization safety data. Vaccine.

[21] Bahri R.A., Abianeh F.E., Peisepar M. (2024). Anosmia or ageusia following COVID-19 vaccination: a systematic review. Ear Nose Throat J.

[22] Coelho P., Paula A., Martins I.V. (2022). Combined central and peripheral demyelination after COVID-19 vaccination. J Neurol.

[23] Arabzadeh Bahri R., Esmaeilpur Abianeh F., Peisepar M. (2024). Anosmia or ageusia following COVID-19 vaccination: a systematic review. Ear Nose Throat J.

[24] European Medicines Agency (EMA). COVID-19 vaccine safety update COMIRNATY BioNTech Manufacturing GmbH. 2021 [Accessed 10 March 2025]. Available from:https://www.ema.europa.eu/en/documents/covid-19-vaccine-safety-update/covid-19-vaccine-safety-update-comirnaty-6-october-2021_en.pdf

[25] Riad A., Pokorná A., Klugarová J. (2021). Side effects of mRNA-based COVID-19 vaccines among young adults (18–30 years old): an independent post-marketing study. Pharmaceuticals.

[26] Shafiee A., Amini M.J., Arabzadeh Bahri R. (2023). Herpesviruses reactivation following COVID-19 vaccination: a systematic review and meta-analysis. Eur J Med Res.

[27] Boas RJEV, de Sousa M.S., Sousa L.F. (2024). Oral herpes zoster following anti-COVID-19 vaccination: is there a relationship?. Oral Surg Oral Med Oral Pathol Oral Radiol.

[28] Akpandak I., Miller D.C., Sun Y., Arnold B.F., Kelly J.D., Acharya NR. (2022). Assessment of herpes zoster risk among recipients of COVID-19 vaccine. JAMA Netw Open.

[29] Pala E., Bayraktar M., Calp R. (2024). The potential association between herpes zoster and COVID-19 vaccination. Heliyon.

[30] Wojcicki A.V., O’Flynn O’Brien K.L. (2021). Vulvar aphthous ulcer in an adolescent after Pfizer-BioNTech (BNT162b2) COVID-19 vaccination. J Pediatr Adolesc Gynecol.

[31] Maeda K., Yamashita D., Takenobu T. (2022). Ulcers on the bilateral palate mucosa following mRNA-based vaccination for coronavirus disease 2019 (COVID-19): a case report. J Stomatol Oral Maxillofac Surg.

[32] Kim H.K., Kim ME. (2022). A case of aphthous stomatitis in a healthy adult following COVID-19 vaccination: clinical reasoning. J Oral Med Pain.

[33] Deng J., Guo H., Kong R., Gao J. (2024). Case report: PFAPA (periodic fever, aphthous stomatitis, pharyngitis and cervical adenitis) syndrome with a novel TNFAIP3 mutation. Immun Inflamm Dis.

[34] Gold M.S., Amarasinghe A., Greenhawt M. (2023). Anaphylaxis: revision of the Brighton collaboration case definition. Vaccine.

[35] Whitaker H.J., Hocine M.N., Farrington CP. (2009). The methodology of self-controlled case series studies. Stat Methods Med Res.

[36] Trifirò G., Coloma P.M., Rijnbeek P.R. (2014). Combining multiple healthcare databases for postmarketing drug and vaccine safety surveillance: why and how?. J Intern Med.

[37] Bonaldo G., Noseda R., Ceschi A., Vaccheri A., Motola D. (2020). Evaluation of the safety profile of rotavirus vaccines: a pharmacovigilance analysis on American and European data. Sci Rep..

[38] Anastassopoulou C., Boufidou F., Hatziantoniou S., Vasileiou K., Spanakis N., Tsakris A. (2023). Adverse events of acute nephrotoxicity reported to EudraVigilance and VAERS after COVID-19 vaccination. Vaccine.

[39] Montano D. (2022). Frequency and associations of adverse reactions of COVID-19 vaccines reported to pharmacovigilance systems in the European Union and the United States. Front Public Health..

[40] European Medicines Agency (EMA). Pharmacovigilance: overview. [Accessed 11 March 2025]. Available from: https://www.ema.europa.eu/en/human-regulatory-overview/pharmacovigilance-overview

[41] Statista (2024). COVID-19-impfungen nach Geschlecht.

[42] Duijster J.W., Lieber T., Pacelli S. (2023). Sex-disaggregated outcomes of adverse events after COVID-19 vaccination: a Dutch cohort study and review of the literature. Front Immunol.

[43] Flanagan KL, Fink AL, Plebanski M, Klein SL. Sex and gender differences in the outcomes of vaccination over the life course. 2017 [Accessed 16 November 2021];33:577–99. Available from: https://pubmed.ncbi.nlm.nih.gov/28992436/10.1146/annurev-cellbio-100616-060718 28992436

[44] Rich J.T., Neely J.G., Paniello R.C., Voelker C.C.J., Nussenbaum B., Wang EW. (2010). A practical guide to understanding Kaplan-Meier curves. Otolaryngol Head Neck Surg.

